# New Appliances and Things Medical

**Published:** 1905-07-22

**Authors:** 


### NEW APPLIANCES AND THINGS MEDICAL.

[We shall be glad to receive, at our Office, 28 & 29 Southampton Street, Strand, London, W.C., from the manufacturers, specimens of all new preparations and appliances which may be brought out from time to time.]

### ARGEX FLUID BEEF.

(Argex Fluid Beef Co., London.)

In a former notice in this journal we discussed shortly the chemical composition of beef teas, so called, and mentioned that as they consisted mainly of the extractive material of the meat their nutritive value was low. In contradistinction to these beef teas and meat extracts a number of liquid preparations have been placed on the market, containing not only the extractive and mineral matter of the original meat, but also a large proportion of proteid material, mostly existing in the form of peptones and albumoses. To a number of these substances the name of fluid beef or fluid meat has been given. We have not analysed Argex Fluid Beef, but it presumably belongs to this class of liquid meat preparation. The dose is one teaspoonful, which may be added to a cup of hot water, the result being a refreshing and in the above sense, nutritive beverage. Argex fluid beef is well borne and liked by patients, and is in every way a suitable and convenient form in which to supply proteid nourishment.

### KELPION (UNG. IODI).

(Kelpin, Limited, 59a Bishopsgate Street Within, E.C.)

Iodine is a medicament which we constantly wish to use, and is best applied locally. Its counter-irritant and antiseptic action are well known and constantly made use of. Two disadvantages attending the use of ordinary preparations of it, however, are the fact that it stains the skin, and is apt to cause too much superficial irritation. There are, nevertheless, certain preparations of iodine on the market which are colourless, but there is some doubt as to whether the iodine in these preparations is sufficiently labile to exert its pharmacological action. The preparation before us does not stain the skin and does not cause much superficial irritation, although it is a strong preparation of iodine, containing 5 per cent.: the B.P. ointment contains 4 per cent. Kelpion must be rubbed on the skin until the colour of the preparation disappears. It differs, too, from most other forms of iodine used therapeutically, in that it contains no iodide of potash. Kelpion consists of a loose compound of iodine with certain heavy hydrocarbon oils. By rubbing this substance on the skin the iodine is liberated and absorbed and the hydrocarbon oil base is left behind and acts as an emollient.

